# Musculoskeletal Lower Limb Injury Risk in Army Populations

**DOI:** 10.1186/s40798-016-0046-z

**Published:** 2016-04-29

**Authors:** Kimberley A. Andersen, Paul N. Grimshaw, Richard M. Kelso, David J. Bentley

**Affiliations:** 1 School of Mechanical Engineering, University of Adelaide, Adelaide, South Australia 5005 Australia; 2 School of Health Sciences, Flinders University, GPO Box 2100, Adelaide, South Australia 5001 Australia

## Abstract

Injuries are common within military populations, with high incidence rates well established in the literature. Injuries cause a substantial number of working days lost, a significant cost through compensation claims and an increased risk of attrition. In an effort to address this, a considerable amount of research has gone into identifying the most prevalent types of injury and their associated risk factors. Collective evidence suggests that training and equipment contribute to a large proportion of the injuries sustained. In particular, the large loads borne by soldiers, the high intensity training programs and the influence of footwear have been identified as significant causative factors of lower limb injury in military populations. A number of preventative strategies have been developed within military bodies around the world to address these issues. The relative success of these strategies is highly variable; however, with advancements in technology, new approaches will become available and existing strategies may become more effective.

## Key Points

- Injury rates in the military are still a significant issue despite substantial research on the topic.
- Injury prevention strategies should be tailored such that training is not negatively affected and may vary by gender.
- Alterations to footwear may have a significant effect on injury epidemiology in the lower limb.

## Review

### Introduction

Military personnel serve in many capacities around the world and there are two factors of paramount importance to all efforts; that soldiers are physically capable for duty and that they return safely. The most prevalent factor that could prevent the achievement of these two criteria is musculoskeletal injury [1–3]. Soldiers injured in basic training may be unable to deploy, while soldiers injured during deployment may not be fit to return to active duty. Furthermore, once a musculoskeletal injury is incurred, the risk of sustaining another such injury increases [3–5] causing a greater risk of attrition. One study found a 13 % increase in the incidence of lower limb injury if the recruit had suffered a previous ankle sprain [4]. While certainly undesirable, some amount of catastrophic injury is unavoidable during deployment and is a direct result of the conditions in which military personnel work. There is, however, a large proportion of injuries that may result from (or be exacerbated by) controllable factors such as training [6–11] and equipment [8, 12, 13].

Substantial research has assessed the risk factors for injury in the military [1, 3, 4, 6, 10, 12, 14]; however, results are often contradictory and focus on individual factors, when in reality, a large number are inextricably linked. Load carriage and training are commonly cited as causative factors towards musculoskeletal injury within military forces, particularly army [1, 3, 4, 6, 8–10, 15–19]; however, the effect that footwear can have on injuries has received minimal attention in previous reviews of military injuries despite the research completed in this area [3, 7, 9, 20–22]. Footwear can have a significant effect on gait and performance of tasks, which in turn can lead to injury. The aim of this review is twofold; first, to review the current literature regarding injury causes in the military and second, to demonstrate the influence footwear can have on injury incidence. This review will investigate the prevalence and risk factors for musculoskeletal injury in army populations, the effects of load carriage, training and footwear and finally, schemes for injury prevention and their effectiveness.

### Methodology

This review is a narrative review and the authors do not claim to have analysed all of the available literature; however, they are confident that an accurate snapshot of the current state of the literature has been provided. Articles were sourced from Google Scholar, Pubmed and the Defense Technical Information Center using the search terms ‘military musculoskeletal injury’, ‘load carriage’, ‘military footwear’ and ‘combat boots’. The reference lists of the articles found were then searched for other relevant articles not identified in the initial search.

### Injuries and Associated Factors

#### Injury Rates and Definitions

Injury can affect the combat readiness of any soldier. Therefore, it is important to consider the rates at which injuries occur and in what circumstances. There is significant variation between reported injury rates from different sources (Table 1), and this is largely attributable to the data and definitions used, the nature of the study and the injury types included in the studies. Injury definitions are highly dependent on the data source. A common definition of injury is anything resulting in a medical visit [1, 4, 6, 12]. This definition is particularly common for retrospective studies, where database information is used as the source data [6, 12]. An extension to this definition is to search for specific injury codes [14]. Prospective studies can potentially result in higher recorded injury rates due to the increased awareness of injury amongst participants [4, 10]. These studies may also define injury based on specific examination results [10]. The injury types included can also alter results; some studies neglect cutaneous injuries [1] while others only include a particular injury [7, 8, 10, 14]. For example, a recent study on the United States (US) Army [14] only reported on stress fractures.

**Table 1:** Injury rates amongst military populations

Author (s)	Year	Rate [% (*n*)]	Observation period (weeks)	Study population	Injuries studied	Source data
Milgrom et al. [10]	1985	31 (91)	14	Male IDF recruits (*N* = 295)	Stress fracture	Empirical data
Jones et al. [4]	1993	45.9 (139)	12	Male US Army infantry recruits (*N* = 303)	All injuries—lower limb only	Empirical data
Almeida et al. [1]	1999	39.6 (482)	12	Male US Marine recruits (*N* = 1296)	All injuries	Empirical data
Defence Health Service Branch [12]	2000	9.1 (5038)	52	Full-time ADF personnel (*N* = 55,574)	All injuries	DEFCARE data (1997/98 financial year)
		3.9 (1067)	52	Part-time ADF personnel (*N* = 27,027)		
Davidson et al. [6]	2008	26.7 (2575)	48 (11 months)	Active NZDF personnel (*N* ≈ 10,500)	All injuries—lower limb only	ACC claim forms
Knapik et al. [14]	2012	1.93 (9182)	520 (10 years)	Male US Army recruits (*N* = 475,745)	Stress fracture	DMDC Master Personnel File, DMSS, and MEPS database (1997–2007)
		7.99 (8622)	520 (10 years)	Female US Army recruits (*N* = 107,906)		

*ACC* Accident Compensation Corporation; *ADF* Australian Defence Force; *DMDC* Defense Manpower Data Center; *DMSS* Defense Medical Surveillance System; *IDF* Israeli Defence Force; *MEPS* Medical Entrance Processing Station; *NZDF* New Zealand Defence Force; *US* United States

Two studies revealed a significant injury trend within the military services, with army personnel sustaining at least twice as many injuries as the navy and air force in the New Zealand Defence Force (NZDF) [6] and accounting for 71 % of the total cost of workers compensation claims in the Australian Defence Force (ADF) [12]. This risk is attributed to the greater proportion of time spent training in the field [6]. Another important finding was that part-time personnel [12] and recruits [6] were at significantly greater risk of injury than full-time personnel.

#### Types of Injury

The most common type of military injuries reported are sprains, particularly ankle sprains [1, 3, 4, 6, 12]. Other common injuries sustained in military populations are medial tibial stress syndrome [1, 3, 12], patellofemoral syndrome [4, 12], lower back pain [1, 3, 23], tendinitis [1, 3, 4, 12, 23], stress fractures [1, 3, 4, 6, 10, 12, 14, 23] and iliotibial band syndrome [1, 3, 12]. The majority of these injuries are classified as overuse injuries, where the underlying pathology worsens with repeated stress. Almeida et al. [1] found that 78 % of all injuries during physical training were overuse injuries. This is in contrast to the work by Davidson et al. [6] who found that the most common injuries were acute or rapid onset, where the injury could be attributed to a single traumatic event. It should, however, be noted that Davidson et al. [6] captured data from the entire New Zealand Defence force and included sporting injuries in their data which may have biased results in favour of acute injuries. Injury types would be expected to vary based on the training undertaken, the role of the unit and by extension the service and country within which the soldier serves.

A study investigating the Israeli Defence Force (IDF) found that they had a high incidence of stress fractures amongst their recruits [10]. This stress fracture incidence is similar to the total injury occurrence obtained in other studies, and the authors partially attributed this to the prospective nature of the study. Other injury types were not reported in this study; however, other studies on the IDF that have included other injuries have also noted a high frequency of stress fractures in comparison to other injuries [22, 23]. This suggests the possibility that IDF training practices may cause an abnormally high risk of stress fracture.

#### Injury Location

The majority of injuries occur in the lower limb, with one study [1] reporting up to 82 % of injuries in physical training occurring in this region. Another study conducted on the Australian Defence Force (ADF) [12] found that 31.7 % of injuries for the ADF population overall and 48 % of the injuries sustained during physical training occurred in the lower limb. The prevalence of injury in this region during military training is high enough to warrant studies be conducted that focus solely on the injury epidemiology of the lower limb [4, 6, 10].

#### Risk Factors

Many studies have also delved into probable causes for injuries and risk factors associated with their development. Injury causes nominated in the literature include high volumes of vigorous physical training [1, 3], high running mileage [1, 3, 4, 6, 8–10, 15], rapid onset of activity at the start of physical training [1, 13, 24] and weight-bearing activities [3, 4, 16–19]. It was previously noted that recruits were at a higher risk of suffering an injury. All recruits will undergo physical training, and the potential injury causes listed are all present during this time, particularly the rapid onset and volume of physical training, thus explaining the greater risk of injury to recruits [6, 25].

Identified risk factors for injury during physical training are history of inactivity [3, 4, 6, 24, 26, 27], previous injury history [3, 4, 17], smoking [2–4], age [2–4], body-mass index (BMI) [2, 14, 23, 27], flexibility [4, 12], other anthropometric factors [3, 9, 12, 14, 21, 23, 26, 27] and gender [23, 27].

History of inactivity, prior injury history and smoking can increase the risk of injury via a weakening of the musculoskeletal structure compared to someone who is fit and healthy [2–4, 17]. In the case of inactivity, the risk is also related to the sudden elevation in training level resulting in excessive stresses being applied to the body [23]. Anthropometrical characteristics such as high/low foot arches [3, 9, 28], bone geometry [3, 14, 23, 26], genu valgum [3], alignment abnormalities [12] and height [27] have been reported as risk factors for injuries; however, these are intrinsic factors and hence highly individual and difficult to alter in an effort to lower injury rates.

The risk associated with age varies between studies. One study found that recruits over 19 years of age [2] were at greater risk of injury, while another found it to be those over 24 years old [4]. Other studies have, however, made note of younger recruits being at risk of injury in addition to their older counterparts [3, 29]. One such study found that recruits under the age of 19 and over the age of 23 were at greater risk of attrition than those between [29]. Given that the ages mentioned are relatively young and similar, the makeup of the sample group and how finely the ages are separated may significantly affect the determination of risk factors associated with age [29].

High and low BMI have been identified as risk factors for musculoskeletal injury [2, 14, 23, 27]. It has been postulated that a lower BMI may reflect a deficiency in bone mass, thus increasing the risk of injury as they may lack the muscle mass or bone strength to effectively perform certain tasks, resulting in overexertion of the risk tissues [14]. High BMI as a risk factor is difficult to assess due to the nature of the measure. As BMI is calculated based on a person’s mass divided by their height squared, it does not take into account their percentage body fat. Excess body fat has been identified as a risk factor for injury [19, 27] in men but not for women [27].

The effect of flexibility on injury has been found to observe a U-shaped curve in that both extremes of flexibility indicate an elevated risk [4]. Low flexibility can restrict movement in certain tasks and may result in an increase in muscular strains as the body seeks to overcome this restriction [4]. High flexibility has been associated with increased joint laxity, which can lead to an increase in the frequency of dislocations and sprains [4, 12]. A pre-exercise stretching protocol was trialled in the Australian Army with an experimental (stretching protocol) and control (no stretching) group participating, however, no clinically significant changes to injury risk were found [30].

Gender represents an additional injury risk factor in military personnel. Typically, female recruits have been found to be at greater risk of injury than their male counterparts [23, 27, 31]. It has, however, also been found that female recruits are generally less fit (e.g. lower strength, aerobic capacity or flexibility) at the commencement of physical training [23, 27]. Studies that have sorted male and female recruits by entry-level fitness rather than gender have found recruits with slower 1 mile run times were at greater risk of injury than the faster recruits [27, 32]. This suggests that the risk factor for injury may not be gender per se, but rather low entry level fitness [3, 27, 32]. It should, however, be noted that shorter tibial length [26] and stature [27] have also been identified as risk factors for musculoskeletal injury and female recruits are at greater risk of sustaining a stress fracture than male recruits [27, 31].

### Load Carriage

Weight-bearing activities have been reported as a potential cause for injury amongst military populations [3, 4, 16–19]. This is largely attributable to the amount of equipment and body armour that soldiers carry, whether in training or during operations. The energetic cost associated with load carriage is also of interest as increases in soldier fatigue have been found to cause a decrease in cognitive function and information processing and an increase in injury risk [16, 19, 33, 34].

In the *US Army Field Manual 21-18* [35], combat load is divided into three categories; fighting load (FL), approach march load (AML) and emergency approach march load (EAML). Fighting load consists of only the weapons and ammunition required to complete the objective, while AML is the equipment and munitions required to fight and exist until resupply. These should not exceed 21.7 and 32.7 kg, respectively. Emergency approach march load is used when other methods of transportation are unavailable. Soldiers may be required to carry up to 54.5 kg for several days, covering 20 km/day, with masses up to 68 kg being feasible.

A study conducted by Orr et al. [34] found that, on average, soldiers in the ADF carried 28.4 kg while in patrol order and 56.7 kg in marching order. Despite the difference in terminology, it would be reasonable to assume that patrol order and marching order are comparable to FL and AML, respectively. If this is the case, both orders exceed the recommended load limit set by the US Army Field Manual.

One study following a US Army regiment on deployment noted the loads carried by each of the 29 soldier positions within the unit for each of the three load categories [33]. No position carried a FL or AML that was lower than the respective limits, on average carrying 28.6 and 46.0 kg in the two categories. The EAML’s carried were all under the upper limit of 68 kg. It should, however, be noted that three positions were carrying over 90 % of their body weight (BW), the highest being 98.8 % BW. The maximum load that a soldier should carry has been reported as 45 % BW [19], and soldiers were carrying more than this in both AML and EAML representing a substantial risk of injury [33].

With such high loads, the method of load carriage is of major importance. For infantry, the most efficient place to carry load is on the torso with the use of a pack system [16]. In an effort to balance the moment caused by the loaded pack, soldiers will increase the forward tilt of their trunk, placing increased load on the lumbar spine [16]. Load carriage vests may be employed to lower the energetic cost, improve posture and hence lower the risk of injury by redistributing weight forwards and reducing the forward tilt [16]. Other factors related to the use of packs are the rigidity of the pack frame [36], location of load in the pack [16], terrain [16] and gender effects [16]. In questionnaires, women have been found to comment more than men about uncomfortable pack straps, poorly fitted pistol belts and unstable rucksacks, suggesting that current pack systems do not adequately cater for the differing anthropometry between the two genders [16].

Increases in the load carried, travel speed or gradient of the terrain will cause an increase in energetic cost [16, 17, 37]. According to Orr [17], an increase in speed of 0.5 km/h or gradient of 1 % is equivalent to an increase in mass of 10 kg. It has been shown that amongst fit individuals walking at a given speed and gradient, the energy cost of carrying up to 30 % BW as external weight is the same as if that weight were additional BW [19]. After this limit, increases in the energetic cost of load carriage are larger than increases in load, hence diminishing the potential benefits of carrying the additional load.

Injuries associated with load carriage are foot blisters [16, 19, 38, 39], lower back pain [16, 19, 39], metatarsalgia [16, 19], stress fractures [16, 19, 39], knee pain [16, 19, 39], rucksack palsy [16, 19, 33, 39], sensory neuropathies [16, 39] and local discomfort [19, 33]. A large proportion of these injuries are due to the increase in load due to the soldiers pack and equipment. For example, foot blisters are common due to the increase in pressure on the plantar surface of the foot and braking forces during locomotion [16]. Similarly, lower back pain, metatarsalgia, stress fractures and knee pain are due to increases in load and the kinematic adjustments to compensate for it [16].

Rucksack palsy is a traction injury of the upper brachial plexus that is caused by pressure from the shoulder straps [16, 39]. This condition can cause numbness, paralysis, cramping and minor pain in the shoulder girdle, elbow flexors and wrist extensors [16], which can severely limit soldier functionality. Excessive load and incorrectly adjusted shoulder straps have been cited as potential causative factors for rucksack palsy [16, 39]. Hip belts are used to alleviate this by distributing load from the shoulders to the hips [16, 39].

### Training

Another aspect of military life that has a significant impact on injuries is the training that personnel must undergo [1, 3, 13, 24]. All recruits typically need to complete some level of physical training or basic combat training before progressing to unit or division specific training programs.

In the previous section on injuries, the most common risk factor for injury was low entry-level fitness [3, 4, 6, 24, 26, 27]. In particular, the shorter, lighter and weaker (as measured using isometric knee and hip extension strength tests) recruits are more likely to suffer a musculoskeletal injury [24]. Despite female recruits typically being smaller and weaker than male recruits, there was no discernible effect of gender in a study by Beck et al. [24]. The sample group used in the study (68 male, 13 female) may account for this result, as while the sample size emulates the percentage of female participants within the Australian Army (16 % in the study compared to 10 % in the Army [39]), there may not have been sufficient statistical power to detect any effect due to the low number of subjects.

It is important that soldiers train, improve and maintain a certain level of physical capacity for any tasks they may need to perform. Some training principles that have been identified are specificity [11, 16], recovery [11, 40] and progressive overload [41].

Training specificity comprises the need to conduct task specific physical conditioning. For example, from a load carriage viewpoint, this means training while carrying loads on a sufficiently regular basis. It should be noted that conflicting data was reported for optimal frequency of training; however, there is some evidence that, after a certain point, fitness gains were reduced with increased exposure to training [11].

**Fig. 1:**
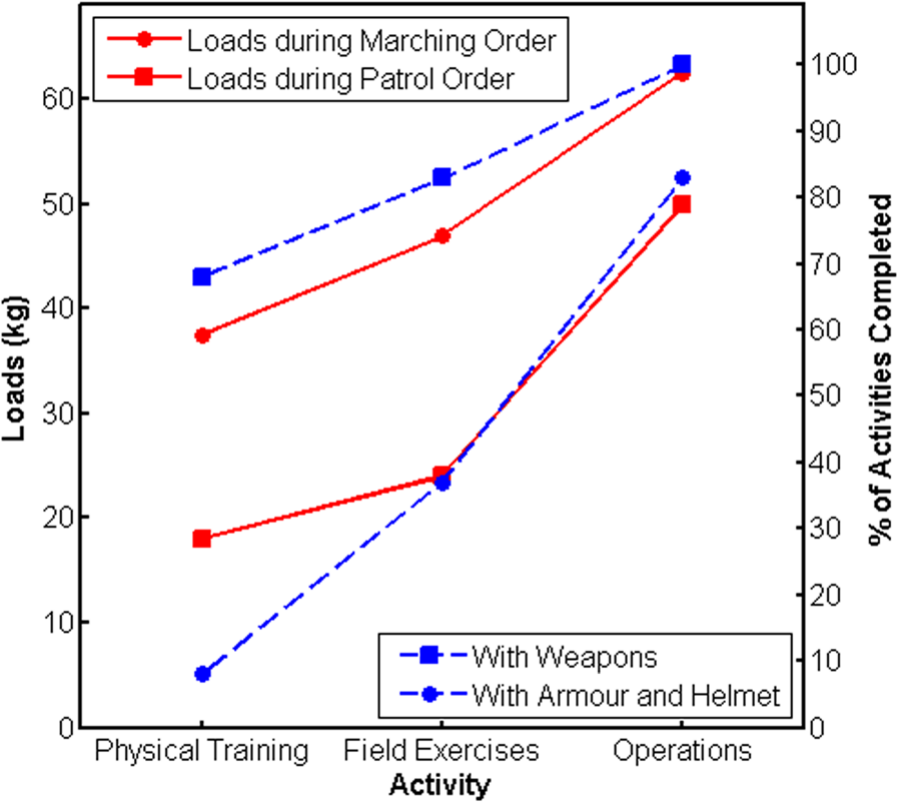
Loads and equipment carried in the Australian Army by activity type. The *solid red lines* indicate the loads carried measured in kilograms. The *dotted blue lines* indicate the percentage of the activities completed with weapons, and armour and helmets in addition to marching and patrol orders. Reproduced from Orr, [17] with permission

It is also important that training intensity matches or builds up to equal those expected during unit operations [16]. In a study by Orr [17], it was found that soldiers were training with as little as half the load used on operation during physical training and field exercises (Fig. 1). Additionally, soldiers were also not training with all the equipment they required on operations (Fig. 1). This represents a significant training error, where a training error is behaviour that results in a sudden increase in the loads applied to the body [13]. Training needs to stress the body sufficiently to elicit a training response; however; with increased intensity, there is also an increased risk of injury [11]. A study by Almeida et al. [1] found that there were more injuries in the weeks involving vigorous training and suggested this was related to cumulative effects over the previous weeks.

The second principle is that of recovery. The body needs to have sufficient recovery from the training program to minimise residual fatigue [41] and prevent overtraining and injury [11, 40]. An important factor to note is that lengthy breaks in conditioning can increase the risk of injury when returning to demanding tasks [1].

A similar concept was noted by Booth et al. [40], who found that recruits participating in a 45-day physical training course showed symptoms of overtraining. These symptoms were not substantial enough to cause a significant decrease in performance. The authors did, however, still recommend more rest and personal time be given to recruits to alleviate the majority of the symptoms. The symptoms presented by the recruits were sleep disturbance, mental fatigue, decreased friendliness, high levels of confusion, negative hormonal changes and increases in muscle inflammation.

The last principle is that of progressive overload, which describes the gradual increase in stress placed on the body during training [41]. It has been shown that the adaptive processes of the body will be required to meet higher physiological demands [1, 41]. This gradual increase may be achieved by altering the intensity and frequency of training, and decreasing the rest periods between training exercises [41]. There is a risk associated with the rapid onset of physical activity [1, 13, 24] suggesting that the initial weeks of training may be too strenuous.

Part-time personnel have been identified as being at high risk of injury, and a potential explanation for this may be related to the training practices of full-time versus part-time personnel [12]. It is likely that part-time personnel spend longer away from training than their full-time counterparts and less time participating in physical training when not engaged in military work [12], thus increasing their injury risk upon returning to training and potentially decreasing the effectiveness of progressive overload.

### Footwear

One of the most important influences on injury mechanics and kinematics of the lower limb is footwear [15, 42, 43]. Of particular interest is the effect footwear can have on gait as any change in gait pattern from what the body is accustomed to is associated with an increased risk of injury [44]. Military footwear, such as combat boots are designed to protect the foot [3, 45, 46], attenuate shock at foot strike [3, 45, 46] and control medio-lateral foot motion [45, 46]. Military footwear, however, has a number of design properties that may result in undesirable effects.

The property with the most obvious effect is the mass of the boots. By wearing boots, the effective mass of the foot is increased [16, 37, 47], thereby increasing the rotational inertia of the leg [16]. This increases the muscle load, energetic cost of locomotion and rate of fatigue and hence increases the risk of injury [47]. The increase in energetic cost for carrying loads on the feet has been found to be four times more costly than walking without load [37].

Footwear can also have a significant influence on gait by restricting motion of the foot [37, 46, 48–53]. This restriction can result in increased loading at the ankle, knee and hip as well as decreased energy absorption during certain parts of the stance phase [48, 49]. This can in turn result in compensatory gait changes [49].

Restriction of the ankle is associated with an increase in energetic cost [37] and is a function of the design of the boot shaft, which provides the primary support in this region. The shaft has two competing design constraints; it must be rigid to support the joint, whilst being flexible enough to allow a sufficient range of motion to achieve efficient locomotion [49]. Even a small change in ankle dorsiflexion can have a significant effect on Achilles tendon strain and hence injury [15]. Ankle sprains have been identified as the most common injury [1, 3, 4, 6, 12], suggesting that current boot shaft design and the running shoes used during physical training may provide inadequate support to the ankle. Unfortunately, specific details regarding the type of sprain and method of injury are not available, and combat boot and running shoes usage during physical training varies between countries and services, so insufficiencies in design cannot be determined. One study [49] evaluating boot stiffness in the transverse plane found that a stiffer boot decreased the range of motion and eccentric energy absorption at the ankle, resulting in a compensatory gait change at the knee joint and decreased efficiency of locomotion.

**Fig. 2:**
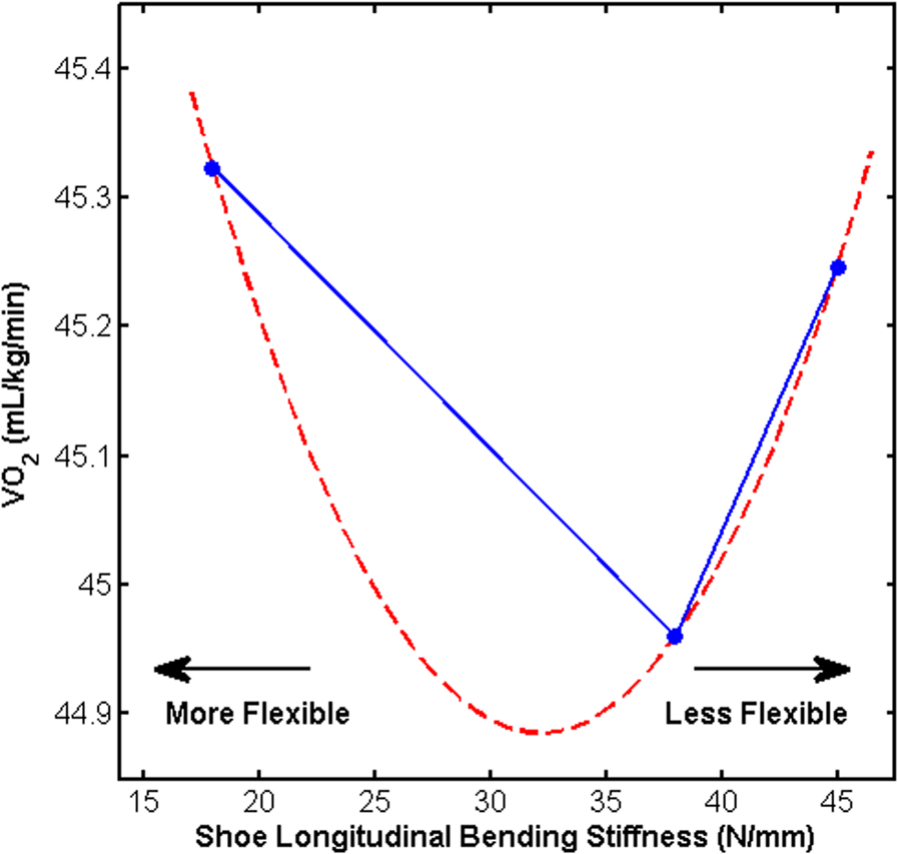
Mean oxygen uptake (VO2) versus shoe forefoot longitudinal bending stiffness. Reproduced from Roy and Stefanyshyn, [53] with permission. The *blue solid line* is the data presented by Roy and Stefanyshyn [53], while the *red dotted line* is a potential trend of energetic cost with shoe longitudinal bending stiffness

A study examining boot stiffness throughout the stance phase concluded that the primary effects of boot stiffness are limited to the ankle [50]. This is however in contrast to other work suggesting sole stiffness plays a significant role at the metatarsophalangeal (MTP) joint [45, 48, 51]. To highlight this fact, when the Swedish Army changed from a M59 combat boot to a M90 combat boot with a more flexible sole under the MTP joint, there was a subsequent increase in stress fractures of the second metatarsal [48]. It was found that the cause of injury was an increase in dorsal metatarsal tension when walking in the M90 boot, potentially due to inadequate support in the forefoot region as a result of the increased flexibility [48]. With respect to energetic cost, it has been shown that optimal forefoot stiffness may be given by a U-shaped curve (Fig. 2), although subject mass had a significant effect on an individual’s optimal stiffness [53].

Hamill and Bensel [45, 46, 54] concluded that soldiers would benefit from an increase in boot sole flexibility; however, they did not test such boots, so a similar response to the introduction of the Swedish M90 combat boot may have occurred. It should also be noted that increased stiffness in the forefoot region of military boots was shown to be a function of the uppers and not the sole structure itself when compared to commercial running shoes [45].

Another factor that can have a significant impact on gait is the attenuation of ground reaction forces at foot strike. Appropriately, cushioned footwear redistributes and reduces plantar pressure [13, 15], while inadequate shock absorption will transmit large forces through the body, potentially resulting in injury [13, 15, 45, 46, 55, 56]. These forces can also result in gait changes by the wearer in an attempt to attenuate the force [42, 57]. When running in minimally cushioned shoes with a predominately rearfoot strike, the ankle is less dorsiflexed (i.e. flatter) at heel strike compared to cushioned shoes [57]. Military boots have consistently been associated with poor shock absorption [15, 45, 46, 56]. One study [55] found no significant evidence of a lack of shock absorption when inspecting ground reaction forces; however, the authors suggested that the subjects were subconsciously adapting to the increased loading. In contrast, studies have shown that footwear with cushioned heels may limit proprioception in the foot, thus compromising the ability of the body to adjust to the loads being applied [43, 58].

Only one study to date has compared injury incidence when physical training is conducted in combat boots or in running shoes and they found no historical evidence to support an increase in injuries when wearing boots [59]. It should be noted that the study was comparing studies conducted prior to 1985 to those after, spanning a 22-year period and a number of operational changes are likely to have occurred during that time and since that may confound results [59]. Further research needs to be conducted to assess the findings of the review [59].

### Injury Prevention

Due to the high number of injuries and their associated costs, preventative strategies for physical training exercises are of great importance. A common suggestion to reduce the occurrence of lower limb injuries is to modify the military training programs [3, 7, 8, 60, 61]. It is postulated that by reducing the cumulative marching distance and increasing training intensity gradually, the incidence of training-related injuries may be reduced [3, 7, 8, 60, 61]. During one controlled trial, this concept was applied in conjunction with a strict sleep regime resulting in a 20 % decrease in injury occurrence [7].

Another study followed an IDF light infantry unit (*n* = 135) through a modified basic training course [23]. This unit was primarily comprised of female soldiers (73 %), and despite a reduction in marching and running, the stress fracture incidence amongst the female personnel was equivalent to that of males completing an unmodified basic training course (12.1 %) [23]. In contrast, male personnel participating in the modified course suffered no stress fractures [23].

A modified training course for female recruits was also implemented in the Australian Army [31]. The modified course lowered march speeds, utilised softer march surfaces and lowered total running distance [31]. Stress fracture incidence was compared between female recruits who completed their training in the year prior to the modified courses implementation (*n* = 143), the female recruits who participated in the modified course (*n* = 161), and male recruits who underwent their training at the same time that the modified course was run but without any modifications (*n* = 1093); the groups did not train together [31]. The stress fracture incidence amongst the female recruits dropped from 11.2 to 0.6 % after implementation of the study bringing rates closer to the male incidence of 0.1 % [31]. Differences between country, the modifications to the training and method of determining stress fracture could account for the differences between the two studies.

Mitigating the incidence of ankle sprains during physical training is desirable due to the high frequency of their occurrence. For example, ankle braces have been shown to be beneficial to paratroopers for reducing ankle injuries [62, 63]. When evaluating the potential use of ankle braces by recruits, Davidson et al. [60] deemed them to be unsuitable due to the ongoing cost, the requirement for individual fitting and their ineffectiveness under extreme conditions. Additionally, ankle braces may cause a tighter fit in the boot shaft region and subsequently restrict motion at the ankle joint. Instead, Davidson et al. [60] advocated the use of wobble boards to build ankle stability and strength. Further research into the boot shaft and its influence on the occurrence of ankle sprains is required to address this issue.

In an attempt to address differences in foot shape amongst recruits, Knapik et al. [9, 21] assigned running shoes based on arch height in two separate studies. Neither study, however, yielded a significant decrease in injury rate for any group.

Shock absorbing insoles have received significant attention in an attempt to offer a low cost method of decreasing injuries by improving impact attenuation; however, results are inconclusive as to their effects. Some reviews have concluded that with appropriate materials, insoles may in the future offer a successful intervention [8, 60]. Another study presented a potentially successful insole, although further testing was required before definitive results could be obtained [15]. Finally, a study on the US Army Band successfully trialled a viscoelastic insole with a 74 % reduction in foot pain and 59 % reduction in back pain when insoles were worn for more than 50 % of the time [64]. Conversely, a review of the past 25 years of interventions implemented by the IDF concluded that insoles or modifications to the boot structure would not be effective in reducing injuries [7].

Other strategies include reducing the mass of all items of equipment carried by soldiers [33], using appropriate load carriage training weights [17], including low-impact exercises such as deep water running [61], utilising pre-entry training courses [24, 60] and the introduction of rest weeks during training [8, 40]. Musculoskeletal screening may also be applied to target recruits at particular risk, such as those with lower strength and flexibility.

When considering preventative strategies, it is imperative that they are directed at the primary factors contributing to the risk of musculoskeletal injuries [3]. Physical limits of soldier endurance must be considered to avoid overtraining [3, 11], and interventions should be such that they do not discourage participation in physical activity [61]. Additionally, interventions to minimise a particular injury should not increase injury risk elsewhere [60]. For example, ankle braces have been associated with an increased risk of knee sprain via compensatory changes resulting in increased loading at this joint [60].

## Conclusions

This review has examined the incidence and epidemiology of injuries within military populations, the effect of load carriage, training and footwear on injury and some potential intervention strategies to mitigate injury. While incidence rates are high, it is feasible to minimise a number of injuries through improvements to equipment design and training practices. These prevention strategies should target the underlying causes of injury for maximum effectiveness.

Furthermore, the effect that combat boot design, such as mass, bending stiffness and impact attenuation, can have on gait and injury epidemiology has been discussed. The boot shaft has been identified as an area of interest for future study due to the high incidence of ankle sprains within army populations.
